# Complete chloroplast genome of *Lycium ferocissimum* (Solanaceae), a species native to South Africa

**DOI:** 10.1080/23802359.2020.1715301

**Published:** 2020-01-21

**Authors:** Zhao Li, Xinyi Zhang, Qingde Zhang, Gulbar Yisilam

**Affiliations:** a Key Laboratory of Plant Stress Biology, School of Life Sciences, Henan University, Kaifeng, China;; b Xinjiang Key Laboratory of Special Species Conservation and Regulatory Biology, Key Laboratory of Plant Stress Biology in Arid Land, College of Life Science, Xinjiang Normal University, Urumqi, China

**Keywords:** *Lycium ferocissimum*, chloroplast genome, phylogeny inference

## Abstract

*Lycium ferocissimum*, known as African boxthorn or boxthorn, is a shrub in the Solanaceae family. In this study, we characterized the complete chloroplast (cp) genome sequence of *L. ferocissimum* using genome skimming data. It had a circular mapping molecular with the length of 155,894 bp, with a large single-copy region (LSC, 86,536 bp) and a small single-copy region (SSC, 18,406 bp) separated by a pair of inverted repeats (IRs, 25,476 bp). The cp genome encodes 113 unique genes, consisting of 79 protein-coding genes, 30 tRNA genes, and 4 rRNA genes, with 20 duplicated genes in the IR regions. The phylogenetic analysis indicated that *L. ferocissimum* is sister to the other three *Lycium* species.

Solanaceae are a monophyletic group containing approximately 100 genera and 2500 species (D’Arcy 1991; Olmstead and Bohs 2007). Species of this family occur on all temperate and tropical continents, and many of which are the world’s most important agricultural species, including potatoes, tomatoes, eggplants, tobacco and chili peppers (Olmstead et al. 2008). However, the phylogeny of Solanaceae has remained obscure due to sparse taxonomic sampling and the limited resolving power of the DNA regions studied. *Lycium ferocissimum* Miers, known as African boxthorn or boxthorn, is a shrub in the Solanaceae family. The species is native to Cape Province and Orange Free State in South Africa and has become naturalized in Australia and New Zealand (Roy et al. 2004). In this study, we reported and characterized the complete cp genome of *L. ferocissimum* using genome skimming data. The genome sequence was registered into GenBank with the accession number MN866909.

One *L. ferocissimum* individual was collected from Cape Town (South Africa; 18°26′57.66″E, 30°36′00.34″S) and a voucher specimen (*Pan Li LP174773*) was deposited at the Herbarium of Zhejiang University (HZU). Genomic DNA was extracted from silica-dried leaf tissue using Plant DNAzol Reagent (LifeFeng, Shanghai). The cp genome was reconstructed based on the paired-end library (≤ 800 bp) data which sequenced on an Illumina HiSeq X10 at Beijing Genomics Institute (BGI, Wuhan, China) with *Lycium ruthenicum* (GenBank accession number: MG29825; Yisilam et al. 2018) as a reference. We annotated the cp genome implemented in Geneious R11 (Biomatters, Auckland, New Zealand) according to descriptions of previous studies (Liu et al. 2017, 2018). Phylogenetic tree for 19 complete cp genome sequences of Solanaceae was inferred using the maximum likelihood (ML) method implemented in RAxML-HPC v8.1.11 on the CIPRES cluster (Miller et al. 2010) with *Cressa cretica* and *Ipomoea purpurea* as outgroups.

The cp genome of *L. ferocissimum* was 155,894 bp long comprising a pair of inverted repeat regions (IRs with 25,476 bp) divided by two single-copy regions (LSC with 86,536 bp and SSC with 18,406 bp). The cp genome encoded a total of 133 genes, of which 113 were unique and 20 were duplicated in the IR regions. The 113 unique genes consisted of 79 protein-coding genes, 30 tRNA genes, and 4 rRNA genes. The overall GC content of the total length, LSC, SSC, and IR regions is 37.9%, 35.9%, 32.3% and 43.2%, respectively. Maximum likelihood (ML) analyses showed that a good resolution of the species of Solanaceae with strong support for all the nodes. The phylogenetic tree revealed that the four *Lycium* species formed one clade with full support, and *L. ferocissimum* is sister to the rest species within the genus (Figure 1).

**Figure 1.**
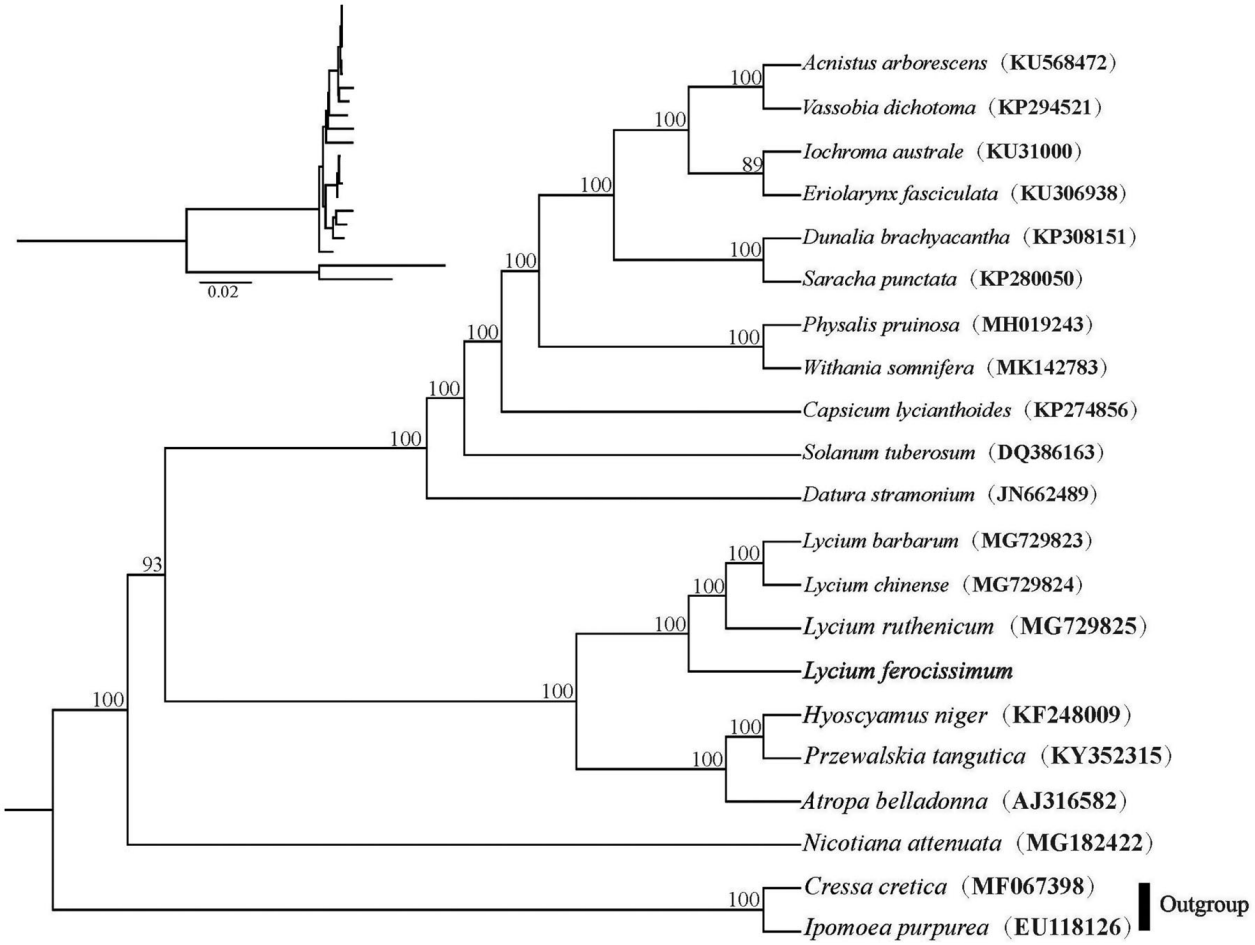
Phylogenetic relationships of Solanaceae inferred based on whole chloroplast genome sequences. Relative branch lengths are indicated at the top-left corner. Numbers above the branches represent bootstrap values from maximum-likelihood analyses.
